# EEG alpha band functional connectivity reveals distinct cortical dynamics for overt and covert emotional face processing

**DOI:** 10.1038/s41598-023-36860-4

**Published:** 2023-06-19

**Authors:** Antonio Maffei, Ambra Coccaro, Fern Jaspers-Fayer, Jennifer Goertzen, Paola Sessa, Mario Liotti

**Affiliations:** 1 Department of Developmental Psychology and Socialisation, University of Padova, Padua, Italy; 2 Padova Neuroscience Center (PNC), University of Padova, Padua, Italy; 3 Department of Psychology, Simon Fraser University, Burnaby, Canada

**Keywords:** Attention, Human behaviour

## Abstract

Current knowledge regarding how the focus of our attention during face processing influences neural responses largely comes from neuroimaging studies reporting on regional brain activations. The present study was designed to add novel insights to this research by studying how attention can differentially impact the way cortical regions interact during emotional face processing. High-density electroencephalogram was recorded in a sample of fifty-two healthy participants during an emotional face processing task. The task required participants to either attend to the expressions (*i.e.*, overt processing) or attend to a perceptual distractor, which rendered the expressions task-irrelevant (*i.e.*, covert processing). Functional connectivity in the alpha band was estimated in source space and modeled using graph theory to quantify whole-brain integration and segregation. Results revealed that overt processing of facial expressions is linked to reduced cortical segregation and increased cortical integration, this latter specifically for negative expressions of fear and sadness. Furthermore, we observed increased communication efficiency during overt processing of negative expressions between the core and the extended face processing systems. Overall, these findings reveal that attention makes the interaction among the nodes involved in face processing more efficient, also uncovering a connectivity signature of the prioritized processing mechanism of negative expressions, that is an increased cross-communication within the nodes of the face processing network.

## Introduction

A rapid and efficient mechanism for face processing, which allows us to derive information about others, is a critical feature of human and non-human primates’ socio-cognitive abilities. Faces are indeed the most important non-verbal cue for social interaction, providing clues and signals that allow us to extract, in a very short time frame, a large variety of information about others, like identity, gender, and ethnicity^1,2^. Most importantly, for the purpose of the present study, we can evaluate others’ emotional expressions, harvesting critical information for inferring others’ affective states.

An interesting aspect of face processing is that, in everyday life, we are constantly exposed to others’ faces and expressions but not all of them are processed equally For instance, when we talk to another person (*i.e.*, a colleague at work), our attention is oriented toward him or her, but most of the time, faces in our environment are processed covertly, without an explicit attentional allocation. Furthermore, it is known at both the behavioral and cortical level that attention is captured differently as a function of the kind of emotion displayed by a face, even though it is not entirely clear if there is an advantage for positive or negative expressions^3–5^. The present research provides novel insights regarding the cortical mechanisms underlying these different modalities of face processing, aiming at shedding new light on the interplay between attention and emotion in shaping brain network organization.

Emotions are generally thought to arise from a combination of bottom-up and top-down influences. Emotional stimuli draw attention rapidly and involuntarily and appear to be processed and encoded automatically^6–9^. This is particularly the case for threatening and aversive stimuli since these types of stimuli are thought to possess adaptive value for survival. For instance, angry faces presented among happy faces are detected faster than vice versa^3^. Simply viewing angry or fearful faces has been found to trigger visceral responses such as increased heart rate and sweating^10^. A number of neural markers associated with the processing of emotion cues have even been observed under conditions where the emotional stimuli are task-irrelevant^11,12^, unattended^13^, or presented below conscious awareness^14,15^, suggesting a certain level of automaticity in the detection of emotional cues. However, several established neural markers also appear to be affected by attentional resources (e.g., fMRI activation in the fusiform face area (FFA)^13^; EPN and LPP responses to emotional faces^9,16^). While several streams of evidence point to the powerful nature of affective cues in automatically capturing processing resources, emotion processing is not solely a bottom-up process. Attention towards emotional content can occur voluntarily and affective cues may be processed consciously in a top-down fashion. The explicit processing of affective cues plays a significant role in social communication and understanding. Top-down control mechanisms can also impact how environmental signals are interpreted and may help determine whether further processing is needed. For example, contextual cues such as positive or negative labels presented before seeing potentially ambiguous facial expressions (e.g., surprised faces) have been found to bias amygdala activations in the direction of the cues^17^. Similarly, subjective belief that a masked emotional face was present in a white noise context resulted in amplification of various emotion-specific components (*i.e.* amygdala activations^18^; N170 component^19^; EPN and LPP components^20^).

Previous electrophysiological studies that tried to characterize the differences between overt and covert emotional face processing almost exclusively focused on event-related potentials (ERPs). Specifically, they investigated either ERP components typically associated with face processing, such as the N170 and the early posterior negativity (EPN), and ERPs associated with processing of motivationally relevant information, such as the late positive potential (LPP). Overall, this line of research revealed that overt face processing is linked with enhanced cortical activity, compared to covert processing^9,21,22^. Furthermore, these studies have found that when attention is directly oriented toward the face, emotional expressions prompt larger responses compared to neutral faces^9,21,22^.

**Figure 1:**
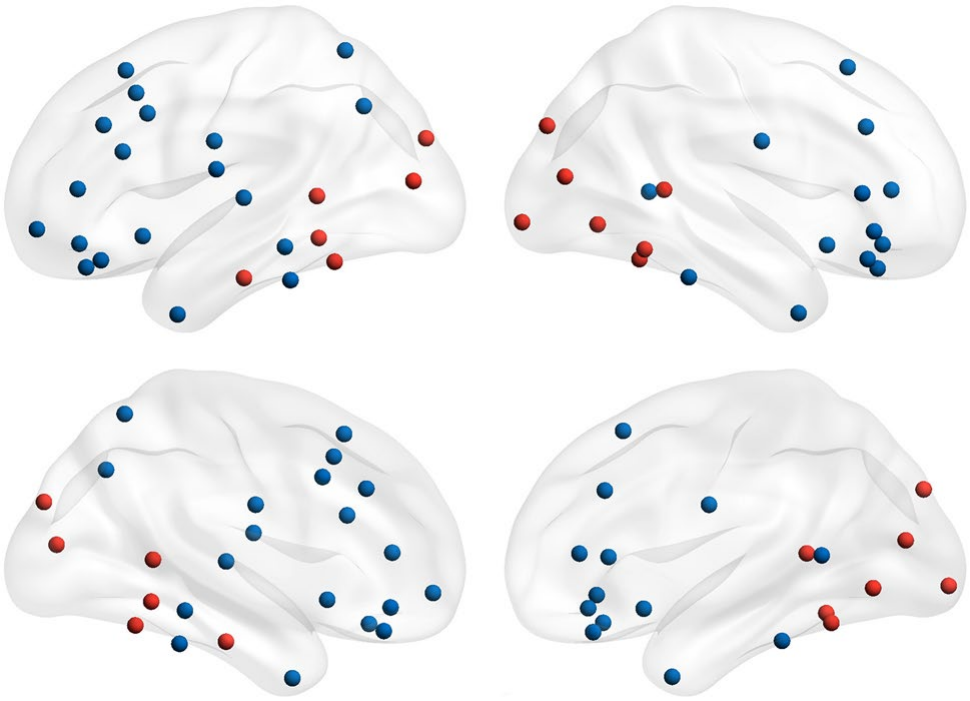
Cortical representation of the Face Processing Network and its two subsystems. Regions belonging to the Core System are identified by the red nodes. Regions belonging to the Extended system are identified by the blue nodes.

These results are important but limited since ERPs can provide only a very coarse view on cortical activity and do not allow any inference regarding the state of the cortical networks underlying this cognitive activity. Indeed, lesion studies, as well as neuroimaging studies in healthy participants have shown that face processing, in general, does not depend on a single, dedicated brain region. Instead, it depends on the activity of a large number of different areas that, altogether, comprise a face processing network (FPN) ^23–27^. The nodes comprising this network are widely distributed across the cortex, but it is possible to recognize two functionally distinct subnetworks, usually labeled as *core* and *extended* systems (Fig. 1).

The nodes typically included in the *core* portion of the FPN span regions in the occipital and temporal cortices, which are critical for building a holistic perception of a face from its basic visual features^25^. These regions are the fusiform gyrus comprising the Face Fusiform Area (FFA), the occipital gyri comprising the Occipital Face Area (OFA), and the superior temporal sulcus (STS). On the other hand, nodes included in the *extended* part of the network are more distributed, comprising regions in parietal, temporal, and frontal cortices^25^. In this *extended system* are typically included areas such as the superior and inferior frontal gyri, the anterior temporal cortex, the insular cortex, and the secondary sensory and motor cortices.

Neurocognitive models of face processing suggest that it is the recruitment of nodes within this extended subnetwork that allows the brain to extract high-order information conveyed by a face, such as identity or emotional expressions^23,25^. Furthermore, a series of recent studies that examined connectivity at different levels (anatomical, functional derived with fMRI, functional derived with MEG/EEG) have started to shed light on the dynamics of cross-communication between these two subsystems as the key for the efficient functioning of the whole FPN^28–31^.

Thus, the present research aims at contributing to uncovering what are the brain mechanisms underlying the difference between overt and covert emotional face processing, with a focus on cortical network dynamics modeled with graph theory.

To accomplish this goal, we designed a task where participants were presented with a series of emotional expressions with a small colored square superimposed on the nose. The task assigned to the participant was to either identify the expression presented or identify the color of the square. In this way, we sought to manipulate participants’ attention toward either the expression conveyed by the face (e.g., overt processing of the face) or the distractor (e.g., covert processing of the face), while preserving an intact processing of the face in both conditions. In order to probe the effect of different expressions, we employed four different expressions, both positive (*i.e.*, happy), negative (*i.e.*, fearful and sad), and neutral.

To characterize functional connectivity, we measured cortical activity with high-density EEG and estimated source-reconstructed whole brain phase-based communication in the alpha band (8–13 Hz).

We decided to measure functional connectivity from source-reconstructed EEG activity because there is growing recognition of the importance of corroborating fMRI-connectivity with electrophysiological measures^32^. Indeed, fMRI connectivity, compared to connectivity measured with high-density EEG and MEG, is limited in measuring the intrinsic oscillatory dynamics of large-scale brain networks, which is instead critical for cognitive activity.

With regards to the choice of the alpha band, it was motivated by a growing recognition that connectivity estimated in this frequency range overlaps with functional networks estimated with fMRI^33,34^. These large-scale brain networks typically also exhibit a strong relationship with alpha oscillatory activity^35^. Furthermore, alpha phase synchrony appears to be crucial for functional integration between cortical regions located to a relative distance from each other^36,37^. Slower oscillations (< 10 Hz) have a characteristic long-range spread that, recruiting the whole cortex, can inform primarily on the integration processes that occur at very long time scales^38–40^. Faster oscillations in the beta/gamma range, on the other hand, tend to be characterized by a very narrow spread. This means that they are very relevant for studying local synchronization, but suboptimal for characterizing communication and integration among distant regions. Finally, alpha phase synchrony, due to its role in coordinating gamma oscillations^41^, is critical for understanding complex sensory processing in general^38,42,43^, and face processing more specifically^44^. Furthermore, recent studies suggest that during face processing the pattern of cortical connectivity measured in the alpha band undergoes a reconfiguration toward a state of high integration^28,29^. Furthermore, the extraction of high-order information relies on more efficient communication between the nodes of the *core* and the nodes of the *extended* part of the FPN^29^. Finally, alpha band connectivity has been successfully used in previous studies to highlight hemispheric asymmetries in affective processing^45^.

In the present research, we leverage the power of graph theory to characterize topological properties of alpha functional connectivity during emotional face processing. Graph theory has become the gold standard in *network neuroscience* since it provides the mathematical tools to concisely model both large-scale as well as local dynamics within a cortical network^46^. Specifically, we focused on two metrics to model whole-brain topology, namely modularity and global efficiency (GE), and on one metric suited to capture interaction among different sub-networks, namely the routing efficiency (RE).

Modularity is a metric that captures the tendency of nodes within a network to segregate into modules. These modules are characterized by high within-module connectivity and low between-module connectivity^47^. The modular structure of brain networks has been consistently observed across multiple scales of connectivity analysis^47,48^, and has been shown to change as a function of cognitive state (resting vs. active condition) and task demands^28,49^. These changes are believed to index the fluctuation between states in which information is processed in segregated compartments (high modularity) and states in which information is broadcasted to and integrated by the whole network^47,50^. This makes this metric very well-suited to quantify the interplay between integration and segregation in the brain.

Global efficiency is a fine-grained measure of information flow in a network. It is based on the assumption that an efficient network is characterized by an architecture that makes possible an easy exchange of information among its nodes. In the context of functional brain networks, this exchange of information can be modeled as the minimum number of steps needed to connect any two nodes in the network, defined *path length*. Global efficiency increases when the path length is short, suggesting that the nodes in the network are very well integrated and the information flows efficiently^51,52^. When the path length is long, the global efficiency of the network decreases, signaling a topological state of low integration.

Finally, we were interested in characterizing the interaction between different sub-networks hypothesized to be critically involved in the task, namely the interaction between the core and extended systems of the FPN. These interactions were modeled using the *routing efficiency* which is a metric based on the shortest path connecting two nodes. The closer two nodes are in the network (*i.e.*, the number of steps needed to move from one node to the other is low) the higher the routing efficiency between them, indicating that they are efficiently integrated^29,53^.

With regards to these metrics, we hypothesized that when facial expressions were processed with an explicit attentional allocation the whole-brain network would have been in a topological state of increased integration. Thus, we predicted to observe lower modularity and higher global efficiency in the overt vs. covert condition. Furthermore, we expected that the cross-communication between the core and the extended systems would be increased, thus we predicted to observe larger routing efficiency in the overt vs. covert condition.

With regards to the role of the emotional expressions during overt processing, we advanced two alternative predictions. We hypothesized to observe, either an increased integration and stronger communication between subnetworks for all emotional categories, irrespective of their valence, or that these network dynamics would be specific for the negative emotional expressions. The former hypothesis would support the idea that it is the extraction of the emotional feature of a face per se to trigger a change in cortical connectivity during face processing. The latter would instead provide support to the idea that changes in cortical connectivity are dependent on the extraction of cues signaling threat/distress in others^54^.

## Results

### Whole brain connectivity

**Figure 2:**
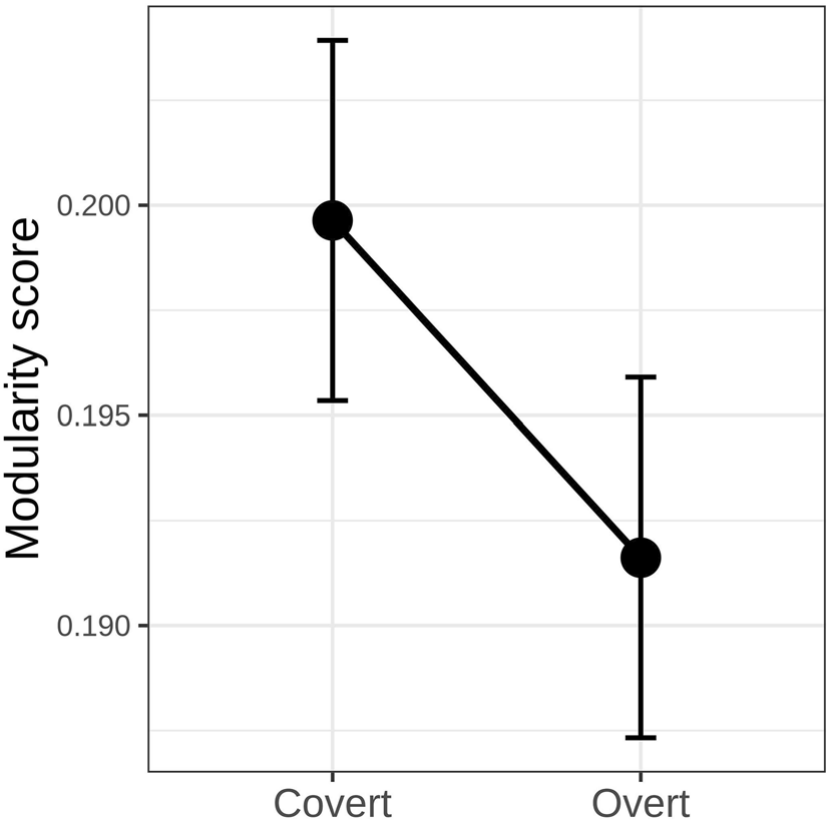
Main effect of the experimental condition on network modularity.

The first aim of this research was to understand how the topology of whole-brain connectivity varies during emotional face processing as a function of attention. The analysis of whole-brain modularity (Fig. 2) revealed a main effect of condition (F_(1,357)_ = 8.05, *p* = 0.004), showing that during overt face processing brain modularity is reduced, thus the network is more integrated, compared to covert face processing. No effect of the emotional expression (F_(1,357)_ = 1.03, *p* = 0.37) nor the interaction (F_(1,357)_ = 0.45, *p* = 0.71) between the two predictors was found.

The analysis of the global efficiency (GE) of the network revealed both a main effect of emotional expression (F_(3,357)_ = 9.2, *p* < 0.001) and a main effect of condition (F_(3,357)_ = 11.05, *p* < 0.001). The analysis also highlighted a significant interaction between the condition and the emotional expression (F_(3,357)_ = 20.05, *p* < 0.001).

**Figure 3:**
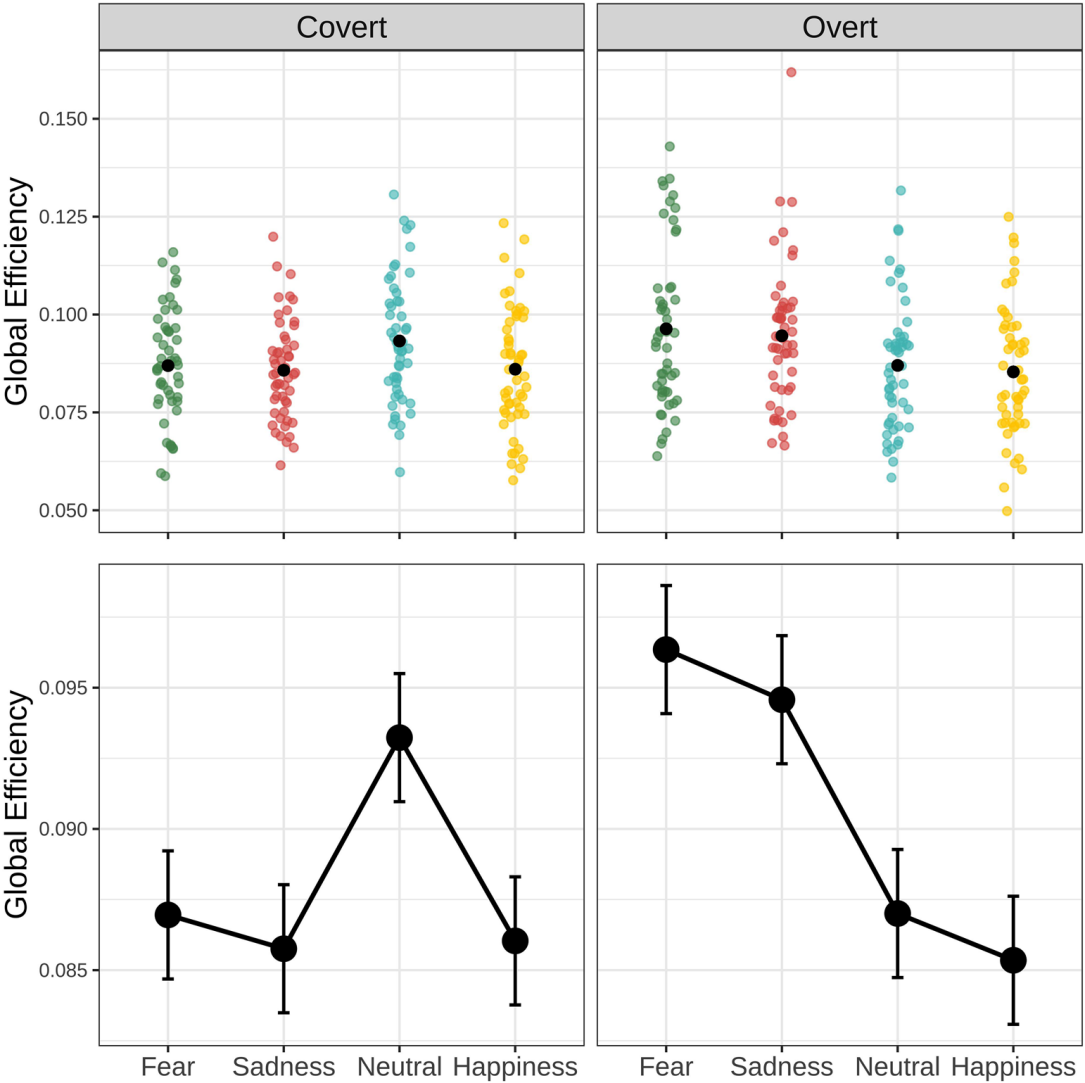
Interaction effect of emotion and experimental condition on network global efficiency. The top panels depict the distribution of individual data points, for each condition and emotional expression. The bottom panels depict the estimated marginal means with their standard errors.

Pairwise contrasts performed on this significant interaction (Fig. 3), revealed that in the overt condition network efficiency was higher for both the expressions of fear and sadness compared to happy and neutral expressions (Fear_overt_ vs. Neutral_overt_ t_(357)_ = 5.5, *p*_*fdr*_ < 0.001; Fear_overt_ vs. Happiness_overt_ t_(357)_ = 6.47, *p*_*fdr*_ < 0.001; Sadness_overt_ vs. Neutral_overt_ t_(357)_ = 4.46, *p*_*fdr*_ < 0.001; Sadness_overt_ vs. Happiness_overt_ t_(357)_ = 5.43, *p*_*fdr*_ < 0.001).

For the covert condition, GE was instead higher specifically during the presentation of neutral expressions compared to all the others (Neutral_covert_ vs. Fear_covert_ t_(357)_ = 3.69, *p*_*fdr*_ < 0.001; Neutral_covert_ vs. Sadness_covert_ t_(357)_ = 4.4, *p*_*fdr*_ < 0.001;Neutral_covert_ vs. Happiness_covert_ t_(357)_ = 4.24, *p*_*fdr*_ < 0.001).

### Functional networks: core and extended face processing networks

The second aim of the present research was to characterize in detail the dynamics within the nodes of the face processing network. Specifically, we focused on the pattern of information routing between the nodes of the core and the nodes of the extended components of the face processing network measured with the routing efficiency (RE). The analysis revealed a significant main effect of emotional expression (F_(3,357)_ = 6.19, *p* < 0.001), and a significant interaction between emotional expression and condition (F_(3,357)_ = 14.09, *p* < 0.001).

**Figure 4:**
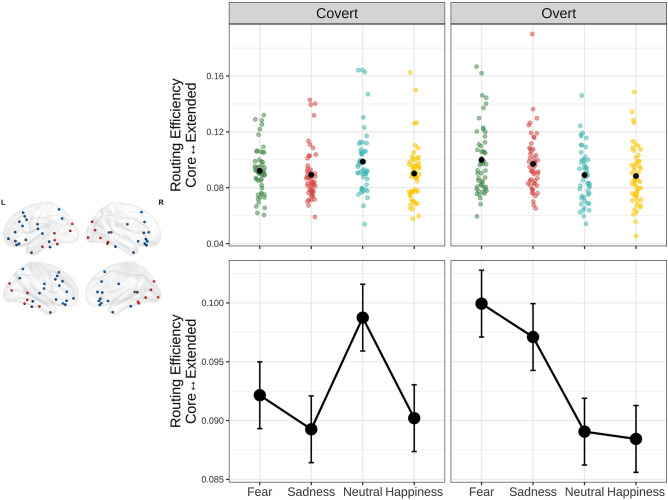
Interaction effect of emotion and experimental condition on the routing efficiency between the Core (red nodes) and the Extended (blue nodes) system of the Face Processing Network. The top panels depict the distribution of individual data points, for each condition and emotional expressions. The bottom panels depict the estimated marginal means with their standard errors.

Pairwise contrasts performed on this significant interaction (Fig. 4) revealed that in the overt condition the efficiency of communication between the two subnetworks was higher for both the expressions of fear and sadness compared to happy and neutral expressions (Fear_overt_ vs. Neutral_overt_ t_(357)_ = 4.8, *p*_*fdr*_ < 0.001; Fear_overt_ vs. Happiness_overt_ t_(357)_ = 5.1, *p*_*fdr*_ < 0.001; Sadness_overt_ vs. Neutral_overt_ t_(357)_ = 3.57, *p*_*fdr*_ < 0.001; Sadness_overt_ vs. Happiness_overt_ t_(357)_ = 3.84, *p*_*fdr*_ < 0.001).

During covert processing RE was instead higher in response to neutral expressions compared to all the other emotions (Neutral_covert_ vs. Fear_covert_ t_(357)_ = 2.93, *p*_*fdr*_ = 0.007; Neutral_covert_ vs. Sadness_covert_ t_(357)_ = 4.21, *p*_*fdr*_ = 0.001; Neutral_covert_ vs. Happiness_covert_ t_(357)_ = 3.79, *p*_*fdr*_ < 0.001).

## Discussion

On a normal day, we encounter dozens of faces, yet we focus our attention on only a small percentage of them, usually in moments during which face-to-face interaction is required. When we focus our attention on a face, we are more likely to extract information relevant for social interactions, such as its emotional expression. On the other hand, it becomes harder to get this information when our attention is focused away from a face. How does the activity in our brain differ between these two different ways of face processing? Answering this question is important to improve our basic understanding of both the brain processes underlying face processing and the mechanisms underlying our ability to use facial information to support social interaction.

The present study sought to contribute to these goals implementing a task in which participants were presented with a series of facial expressions during the recording of their brain activity. Attentional task demands were manipulated by contrasting an emotion categorization task to a perceptual distraction task using identical stimuli but varying instructions. In the overt processing condition, participants had to discriminate the expression of the face presented in the trial, while during the covert processing condition they had to report the color of a square presented overimposed to the face while ignoring the face expression. Brain activity was collected throughout the task with high-density EEG, and a series of functional connectivity metrics were extracted to model the dynamic changes in the patterns of cortical communication. We sought to investigate changes in whole-network connectivity, as well as changes within the distributed network that is involved in face processing. To model this latter pattern of connectivity we first extracted the nodes comprising the *core* and the *extended* systems of the FPN. Nodes in the *core system* were the FFA, OFA, and STS. Nodes in the *extended system* were the superior and inferior frontal gyri, the anterior temporal cortex, the insular cortex, and the secondary sensory and motor cortices. Then, we characterized the efficiency of the information flow between these two systems.

Our analyses revealed a very interesting pattern of results. The first finding was that cortical connectivity is fundamentally different when faces are processed overtly compared to their covert elaboration. The analysis of network modularity suggests that overt processing requires a network topology characterized by lower modularity compared to covert processing. This evidence is in line with a series of recent studies suggesting that the modular architecture of the cortex is not fixed^28,50^. These studies showed that functional connectivity oscillates between states of higher and lower integration, the former indexed by a reduced modularity and the latter by an increased modularity. This dynamic in cortical modularity has been reported both at rest and across different tasks and imaging modalities (fMRI, EEG, MEG), suggesting that it might reflect a fundamental mechanism that supports an optimal allocation of resources in the brain. Our finding is in line with this idea, suggesting that when the environment requires an explicit attentional allocation toward a relevant stimulus, like a face, functional connections in the brain are reshaped to support it.

The second finding of this study is that the different levels of integration observed in cortical connectivity between overt and covert face processing are dependent on the emotion expressed by the face. We initially advanced two alternative predictions, one expecting a modulation of network integration for all emotional faces as opposed to a second expecting that the modulation would be dependent on the valence of the emotions expressed by the faces. The results provide clear support for the latter prediction, suggesting an interaction between valence and attention.

The analysis of the global efficiency of the whole-brain network revealed that, during covert processing, integration is larger only for neutral faces. When attention is, instead, focused on the face, the strongest integration is observed for the two negative expressions of fear and sadness. Furthermore, we found that this pattern was also reflected in the specific dynamics within the two components of the face processing network. During overt processing, we found an improved cross-communication between the nodes of the core and the nodes of the extended system specifically for the two negative expressions. During covert processing we observed, instead, that the efficiency of communication within the face processing network increases only for neutral expressions.

These results are interesting and extend the results of previous neuroimaging studies that showed a similar interaction between attention and valence during emotional face processing^55^. Activation in regions like the face fusiform gyrus (FFA), typically observed in response to faces and usually enhanced by emotional expressions, is highly dependent on where the attentional focus is located. Diverting attention away indeed results in a reduced recruitment of this region. This effect can be observed also in the amygdala, whose activation in response to negative expressions is enhanced by explicit attentional orienting toward the stimulus and suppressed when attention is oriented elsewhere^55^.

This finding also fits with previous literature on the modulation of alpha oscillations during emotional face processing^56^, that points toward an increased power, especially over visual regions, suggesting its involvement in the successful encoding of emotional features, especially for negative expressions.

Here, we extend this evidence by showing that it is not just regional activation, but also the efficiency of functional connectivity mediated by this rhythm, to be critical for processing an emotional face.

When the task required to focus the attention toward the expression we observed that inter-regional communication becomes more efficient for negative expressions, both at the whole-brain level and within the regions primarily involved in face processing. This phenomenon likely reflects the enhanced response of subcortical structures, amygdala in the first place, that, by signaling the relevance of the stimulus in the attentional focus, prompt the observed reshaping of functional connections in the brain. Indeed, amygdala activation has been shown to influence the activation in the nodes of the face processing network, both in the core and extended parts, as shown by investigation of fMRI effective connectivity with dynamic causal modeling^57,58^, as well as in patients with amygdala lesions^59^. Furthermore, due to its wide structural connectivity with the majority of the brain, amygdala has the potential to act as a connector hub, and when activated it can improve the communication between cortical regions^60,61^. Thus, the pattern that we observed on network measures of cortical connectivity can be explained by a recruitment of the amygdala, since it was found only in the experimental condition known to prompt a strong response in this structure, that is negative expressions under overt attentional allocation.

In line with this interpretation, we observed that when attention is diverted away from processing the emotional expression, only neutral faces trigger such dynamics in connectivity. This suggests that without attention, that is in a condition where stimulus saliency detection is known to be blunted^55^, only the prototypical features of a face, conveyed by neutral expressions, have the potential to trigger a modulation of connectivity toward increased efficiency and integration.

An alternative explanation for these latter findings is that the increased integration observed in the covert condition is related to the successful execution of the task of color recognition. We could speculate that the reduced integration found for emotional faces, compared to the neutral ones, depends on the interfering effect exerted by emotional expressions on the processing of the task-relevant feature, that is the color.

Taken together, these results suggest that attention is essential for extracting detailed information from a face, since it prompts a modulation of how the regions involved in this process communicate, rendering it more efficient. Furthermore, they confirm that negative facial expressions benefit from a prioritized processing mechanism, consisting in an increased cross-communication within the nodes of the face processing network, likely mediated by brain structures dedicated to saliency detection. Finally, they reveal that this process is mediated by cortical oscillations in the alpha band, corroborating the growing recognition of alpha waves as an important neural mechanism for distributed cortical processing. Indeed, due to their characteristic spreading, not too wide nor too narrow^38^, alpha oscillations appear critical in integrating a distributed set of regions recruited by a perceptual task, as it happens with emotional face processing.

Finally, it is worth highlighting that our results add to the ongoing discussion about the constructionist nature of emotions^62,63^. This theoretical perspective has been gaining momentum in recent years, suggesting that emotions are a construction of our brain from basic psychobiological mechanisms, rather than resulting from the activity of a set of discrete neural circuits, each dedicated to a specific emotion. Our results add to this view, showing that levels of integration when the brain is processing affective information are not fixed nor specific for each type of emotional information. It rather varies as a function of both attention and affective content. More specifically, we show that these two dimensions interact in shaping the dynamics of cortical communication, and show that the valence of the affective information seems to be a more important feature than its arousal, at least with these kinds of stimuli.

This study and its conclusions do not come without limitations. We first want to acknowledge that the attentional manipulation used in this research is one of many different possible manipulation of attention. Indeed, several studies employed spatial cueing tasks in order to disentangle between covert and overt face processing by manipulating spatial attention. Nevertheless, observing that the findings here reported are in line with these studies employing different manipulations, allows for a good degree of confidence in their interpretation. A second limit of this research is represented by the inherent low anatomical resolution of source reconstructed EEG activity, compared to functional imaging. Thus, we expect that future investigation combining EEG with fMRI will further improve the quantification of cortical connectivity dynamics during emotional face processing.

Finally, we analyzed alpha band cortical oscillatory activity across the entire epoch following face expressions. EEG face studies in the time domain strongly suggest dynamic reconfiguration of neural cortical networks, particularly from early, more automatic stages of face processing (P1,N170) to late, more deliberate stages of emotion face processing (LPP). It would be important, in a future study, to explore whether alpha band functional connectivity varies over time, as a function of attentional demands.

## Methods

### Participants

Fifty-two undergraduate students enrolled in the first- and second-year psychology courses (38 females, age = 19.96 ± 2.97) participated in the study. All participants reported normal or corrected-to-normal visual acuity, normal color vision, no history of neurological and/or psychiatric diseases, no history of drug abuse or learning disabilities. Participants were informed about the study purposes and provided a signed informed consent before beginning the experiment. All the experimental protocols were approved by the Simon Fraser University Research Ethics Board, and all the procedures were carried out in accordance with the principles expressed by the Declaration of Helsinki for human research.

### Experimental task

The experiment used colored photographs of faces taken from the Karolinska stimuli set (Goeleven et al. 2008)^64^ (13 males, 15 females; 4 emotions: fear, sad, happy, and neutral). The faces were displayed on a black background and altered using Photoshop (version 10.0.1, Adobe Inc., San Jose, CA, USA) to obscure the hairline and create identical facial contours. Then each face had a small colored square (red, blue, green, or yellow) superimposed on the nose. Faces were presented for 200 ms, followed by a fixation-cross presented for a randomly jittered interstimulus interval (ISI) of 1700–2300 ms. The stimulus duration was chosen to optimally minimize eye movements that have a latency of at least 180 ms (Itier and Neath-Tavares^16^). The paradigm was coded using Presentation software (Neurobehavioral Systems Inc., Berkeley, CA, USA). The study included two tasks with identical stimuli but different instructions. In the Covert emotion task, participants were asked to attend to the central square irrespective of the surrounding faces, and to choose the color as quickly and accurately as possible (blue, red, green or yellow) by pressing one of four corresponding buttons on a gamepad controller with the index or middle finger of the left or right hand (Logitech, Romanel-sur-Monges, Switzerland). During the Overt emotion task, participants were asked to categorize the expression (happy, fearful, sad or neutral) conveyed by the face, irrespective of the color of the central square, by pressing as quickly and accurately as possible one of the four corresponding buttons on the gamepad controller. To help minimize eye movements, participants were also instructed to keep their eyes on a central fixation cross throughout the whole experiment. Stimuli were displayed in a pseudo-randomized order, constrained so that no more than three stimuli with the same emotion, color, or gender were presented in a row. Participants completed a practice block and were required to reach 80% accuracy before advancing to the actual experiment. Each task (Covert vs. Overt) included 400 stimuli divided into four five-minute blocks separated by short resting periods. To control for potential confounds, button assignments relative to colors (Covert task) or emotions (Overt task) were counterbalanced between participants. The presentation order of the Overt and Covert tasks was also counterbalanced across participants. For each subject, reaction times (RTs) were recorded from stimulus onset and averaged for each combination of condition and emotion. Trials presenting the following characteristics were discarded: RTs shorter than 200 ms (likely corresponded to guesses), excessive eye movements, and RTs longer than 2000 ms (possible button-press errors). Summary statistics of the behavioral performance are reported in Table 1.

**Table 1:** Summary statistics of behavioral performance: Response Times (RTs) and accuracy.

	Happy	Afraid	Sad	Neutral
*RTs in ms*				
Covert	695.73	688.22	687.92	695.77
Overt	783.06	883.34	878.71	800.35
*% Accuracy*				
Covert	95.21	95.39	95.14	95.20
Overt	93.54	73.79	81.80	92.66

### Data acquisition

Data were collected using high-density EEG during the performance of the Covert/Overt face emotion task. Participants sat in a sound-attenuated booth with standardized ambient lighting facing a CRT monitor positioned 60 cm away from the participants’ eyes. The ActiveTwo BioSemi electrode system (BioSemi; Amsterdam, The Netherlands) was used to record continuous EEG from 136 Ag/AgCl electrodes, 130 embedded in an elastic cap and positioned in a modified 10–20 equiradial layout relative to the vertex, including two sensors replacing the “ground” electrodes, *i.e.*, the common mode sense (CMS) active electrode, and the driven right leg (DRL; BioSemi; Amsterdam, The Netherlands). Six additional external electrodes were applied: two at each lateral canthus (HEOG; for horizontal eye movements), two below each eye (VEOG; for vertical eye movements and blinks), and two over each mastoid bone. The continuous signal was acquired with an open pass-band from DC to 150 Hz and digitized at 512 Hz. The amplifier gain was fixed for each active electrode channel at 32 × .

### EEG preprocessing

The preprocessing was performed in Matlab (v2020) using EEGLab (v2021.0) and ERPLab (v8.30) Toolboxes. EEG data were resampled at 256 Hz, re-referenced to the average and a passband filter (0.1–80 Hz) was applied. Bad channels were identified through the plug-in called Clean Rawdata available in EEGLab. The criteria used to exclude a channel were: being correlated less than 0.85 with neighborhood channels and/or if a channel has more line noise than meaningful signal (4 SD). Continuous EEG data were then segmented into epochs starting from -500 ms to 800 ms with respect to stimulus onset. Independent Component Analysis was performed, and artifact components were marked with ICLabel and manually discarded. Additionally, epochs with a peak-to-peak amplitude exceeding ± 100 μV in any channel were identified using a moving window procedure (window size = 200 ms, step size = 20 ms) in order to discard epochs still displaying eye-blinks or movement artifacts. Finally, the average percentage of epochs retained for each condition was 98%.

### EEG source modeling and connectivity analysis

Brainstorm and Matlab were used in the processing phase. With the goal of modeling source activity, a forward model was calculated with the BEM, a three-layer boundary element method, and the source was estimated with the weighted Minimum Norm Estimation (wMNE). This inverse solution was then downsampled to 148 cortical parcels defined by the Destrieux Atlas. Functional connectivity in the alpha band (8–12 Hz) between each parcels pair was estimated with the Phase-Locking Value (PLV), separately for each condition.

The last part of the processing phase employed functions from the Brain Connectivity Toolbox (BCT) in order to estimate several metrics, after thresholding each connectivity matrix retaining the 20% of the strongest connections. The global metrics applied to the whole network (148 vertices) were Global Efficiency and Modularity. Global efficiency is the average inverse shortest path length in the network, computed according to the formula:

$$E_{glob} = \frac{1}{{n\left( {n - 1} \right)}}\mathop \sum \limits_{i \ne j}^{n} \frac{1}{{d_{ij} }}$$

where *n* is the number of nodes in the network and *d*_*ij*_ is the shortest path between node *i* and node *j*.

Modularity represents instead a statistics that quantifies how well a network can be clustered into segregated communities of nodes, according to an algorithm that tries to maximize within-module connection density while minimizing between-modules connection density^65,66^. It is computed according to the formula:

$$Q = \frac{1}{2m} \mathop \sum \limits_{ij}^{{}} \left[ {A_{ij} - \frac{{k_{i} k_{j} }}{2m}} \right] \delta \left( {c_{i} ,c_{j} } \right)$$

where *A*_*ij*_ represents the edge weight between node *i* and node *j*, *k*_*i*_ and *k*_*j*_ represent the weighted degree of nodes *i* and *j*, *m* is the sum of all the edge weights, *c*_*i*_ and *c*_*j*_ are the communities of nodes *i* and *j*, and δ is the Kronecker delta function.

In order to have a fine-grained assessment of integration within the face processing network we used the routing efficiency between the nodes of the *core* (CS) and the nodes of the *extended* (ES) systems comprising the FPN ^29^. This required first the computation, with the Floyd-Warshall algorithm, of the whole-network shortest path length matrix *R*_*mat*_ defined as:

$$R_{mat} = \left( {\begin{array}{*{20}l} 1 \hfill & {\frac{1}{{SPL_{1,2} }}} \hfill & \cdots \hfill & {SPL_{1,n} } \hfill \\ {SPL_{2,1} } \hfill & 1 \hfill & \cdots \hfill & {SPL_{2,n} } \hfill \\ \vdots \hfill & \vdots \hfill & \ddots \hfill & \vdots \hfill \\ {SPL_{n,1} } \hfill & {SPL_{n,1} } \hfill & \cdots \hfill & 1 \hfill \\ \end{array} } \right)$$

in which off-diagonal entries store the inverse of the shortest path length between any pair of nodes in the network. Then, the maximum routing efficiency between a node belonging to the CS and a node belonging to the ES was computed as:

$${R}_{eff}={max\,R}_{mat(CS,ES)}$$

Node assignment to the two subsystems, with their respective coordinates in the MNI space, are provided in the Supplementary Table 1.

### Statistical analysis

Statistical modeling was performed fitting a linear mixed-effect model to the data of each metric. Model specification included, as fixed-effect predictors, the factors Condition (2 levels; covert vs. overt), Emotional expression (4 levels; fear, sad, happy and neutral), and their interaction. In order to model the non-independence of the observation due to the repeated-measurement design we also included a random intercept for each subject. An *F* test was employed to test for significant effect of each fixed-effect predictor, using the Satterthwaite approximation for degrees of freedom. Significant effects were further characterized using pairwise *t*-tests, controlling for the false discovery rate, arising from the multiple comparisons, with the Benjamini and Hochberg’s procedure.

## Supplementary Information

Supplementary Table 1.

## Data Availability

Data and code for replicating the results in the present study can be accessed at https://osf.io/db8qv/?view_only=0f413b6344e647a58a1d9d0ec1b7d1d9. Raw EEG data and EEG preprocessing script are available from the corresponding author upon request.

## References

[1] Dobs K, Isik L, Pantazis D, Kanwisher N (2019). How face perception unfolds over time. Nat. Commun..

[2] Adolphs R, Birmingham E, Calder AJ, Rhodes G, Johnson MH, Haxby JV (2011). Neural substrates of social perception. Oxford Handbook of Face Perception.

[3] Ceccarini F, Caudek C (2013). Anger superiority effect: The importance of dynamic emotional facial expressions. Vis. Cogn..

[4] Eimer M, Kiss M (2007). Attentional capture by task-irrelevant fearful faces is revealed by the N2pc component. Biol. Psychol..

[5] Hodsoll S, Viding E, Lavie N (2011). Attentional capture by irrelevant emotional distractor faces. Emotion.

[6] Holmes A, Nielsen MK, Tipper S, Green S (2009). An electrophysiological investigation into the automaticity of emotional face processing in high versus low trait anxious individuals. Cogn. Affect. Behav. Neurosci..

[7] Ohman A (2005). The role of the amygdala in human fear: automatic detection of threat. Psychoneuroendocrinology.

[8] Phelps EA (2006). Emotion and cognition: insights from studies of the human amygdala. Annu. Rev. Psychol..

[9] Maffei A (2021). Spatiotemporal dynamics of covert versus overt processing of happy, fearful and sad facial expressions. Brain Sci..

[10] Ohman A, Soares JJ (1998). Emotional conditioning to masked stimuli: expectancies for aversive outcomes following nonrecognized fear-relevant stimuli. J. Exp. Psychol. Gen..

[11] Eimer M, Holmes A, McGlone FP (2003). The role of spatial attention in the processing of facial expression: An ERP study of rapid brain responses to six basic emotions. Cogn. Affect. Behav. Neurosci..

[12] Holmes A, Vuilleumier P, Eimer M (2003). The processing of emotional facial expression is gated by spatial attention: Evidence from event-related brain potentials. Brain Res. Cogn. Brain Res..

[13] Vuilleumier P, Armony JL, Driver J, Dolan RJ (2001). Effects of attention and emotion on face processing in the human brain: An event-related fMRI Study. Neuron.

[14] Whalen PJ (1998). Masked presentations of emotional facial expressions modulate amygdala activity without explicit knowledge. J. Neurosci..

[15] Prete G, Capotosto P, Zappasodi F, Laeng B, Tommasi L (2015). The cerebral correlates of subliminal emotions: An electroencephalographic study with emotional hybrid faces. Eur. J. Neurosci..

[16] Itier RJ, Neath-Tavares KN (2017). Effects of task demands on the early neural processing of fearful and happy facial expressions. Brain Res..

[17] Kim H (2004). Contextual modulation of amygdala responsivity to surprised faces. J. Cogn. Neurosci..

[18] Pessoa L, Japee S, Sturman D, Ungerleider LG (2006). Target visibility and visual awareness modulate amygdala responses to fearful faces. Cereb. Cortex.

[19] Wild HA, Busey TA (2004). Seeing faces in the noise: stochastic activity in perceptual regions of the brain may influence the perception of ambiguous stimuli. Psychon. Bull. Rev..

[20] Lee K-Y (2010). Neural correlates of top-down processing in emotion perception: An ERP study of emotional faces in white noise versus noise-alone stimuli. Brain Re.s.

[21] Jaspers-Fayer F (2022). Spatiotemporal dynamics of covert vs. overt emotional face processing in dysphoria. Front. Behav. Neurosci..

[22] Wronka E, Walentowska W (2011). Attention modulates emotional expression processing. Psychophysiology.

[23] Duchaine B, Yovel G (2015). A revised neural framework for face processing. Annu. Rev. Vis. Sci..

[24] Haxby JV, Hoffman EA, Gobbini MI (2000). The distributed human neural system for face perception. Trends Cogn. Sci..

[25] Haxby, J. V. & Gobbini, M. I. Distributed neural systems for face perception. *Oxford Handbook of Face Perception* https://www.oxfordhandbooks.com/view/10.1093/oxfordhb/9780199559053.001.0001/oxfordhb-9780199559053-e-006 (2011) doi:10.1093/oxfordhb/9780199559053.013.0006.

[26] Haxby JV, Hoffman EA, Gobbini MI (2002). Human neural systems for face recognition and social communication. Biol. Psychiatry.

[27] Prete G, Croce P, Zappasodi F, Tommasi L, Capotosto P (2022). Exploring brain activity for positive and negative emotions by means of EEG microstates. Sci. Rep..

[28] Maffei A, Sessa P (2021). Event-related network changes unfold the dynamics of cortical integration during face processing. Psychophysiology.

[29] Maffei A, Sessa P (2021). Time-resolved connectivity reveals the “how” and “when” of brain networks reconfiguration during face processing. Neuroimage Rep..

[30] Wang Y (2020). Multimodal mapping of the face connectome. Nat. Hum. Behav..

[31] Zhao Y, Zhen Z, Liu X, Song Y, Liu J (2018). The neural network for face recognition: Insights from an fMRI study on developmental prosopagnosia. Neuroimage.

[32] Palva S, Palva JM (2012). Discovering oscillatory interaction networks with M/EEG: Challenges and breakthroughs. Trends Cogn. Sci..

[33] Mantini D, Perrucci MG, Del Gratta C, Romani GL, Corbetta M (2007). Electrophysiological signatures of resting state networks in the human brain. Proc. Natl. Acad. Sci..

[34] Samogin J (2020). Frequency-dependent functional connectivity in resting state networks. Hum. Brain Mapp..

[35] Sadaghiani S (2010). Intrinsic connectivity networks, alpha oscillations, and tonic alertness: A simultaneous electroencephalography/functional magnetic resonance imaging study. J. Neurosci..

[36] Muller L, Chavane F, Reynolds J, Sejnowski TJ (2018). Cortical travelling waves: Mechanisms and computational principles. Nat. Rev. Neurosci..

[37] van Driel J, Knapen T, van Es DM, Cohen MX (2014). Interregional alpha-band synchrony supports temporal cross-modal integration. Neuroimage.

[38] Maffei A (2020). Spectrally resolved EEG intersubject correlation reveals distinct cortical oscillatory patterns during free-viewing of affective scenes. Psychophysiology.

[39] Massimini M (2004). The sleep slow oscillation as a traveling wave. J. Neurosci..

[40] Steriade M, McCormick DA, Sejnowski TJ (1993). Thalamocortical oscillations in the sleeping and aroused brain. Science.

[41] Bahramisharif A (2013). Propagating neocortical gamma bursts are coordinated by traveling alpha waves. J. Neurosci..

[42] Jerbi K (2009). Task-related gamma-band dynamics from an intracerebral perspective: Review and implications for surface EEG and MEG. Hum. Brain Mapp..

[43] Maffei A, Polver S, Spironelli C, Angrilli A (2020). EEG gamma activity to emotional movies in individuals with high traits of primary “successful” psychopathy. Brain Cogn..

[44] Rossion B (2014). Understanding face perception by means of human electrophysiology. Trends Cogn. Sci..

[45] Wyczesany M, Capotosto P, Zappasodi F, Prete G (2018). Hemispheric asymmetries and emotions: Evidence from effective connectivity. Neuropsychologia.

[46] Bullmore E, Sporns O (2009). Complex brain networks: Graph theoretical analysis of structural and functional systems. Nat. Rev. Neurosci..

[47] Betzel RF, Fukushima M, He Y, Zuo X-N, Sporns O (2016). Dynamic fluctuations coincide with periods of high and low modularity in resting-state functional brain networks. Neuroimage.

[48] van den Heuvel MP, Sporns O (2013). Network hubs in the human brain. Trends Cogn. Sci..

[49] Di X, Gohel S, Kim E, Biswal B (2013). Task vs. rest—different network configurations between the coactivation and the resting-state brain networks. Front. Human Neurosci..

[50] Betzel RF, Byrge L, Esfahlani FZ, Kennedy DP (2020). Temporal fluctuations in the brain’s modular architecture during movie-watching. Neuroimage.

[51] Achard S, Bullmore E (2007). Efficiency and cost of economical brain functional networks. PLoS Comput. Biol..

[52] Bullmore E, Sporns O (2012). The economy of brain network organization. Nat. Rev. Neurosci..

[53] Avena-Koenigsberger A (2019). A spectrum of routing strategies for brain networks. PLoS Comput. Biol..

[54] Fusar-Poli P (2009). Functional atlas of emotional faces processing: a voxel-based meta-analysis of 105 functional magnetic resonance imaging studies. J. Psychiatry Neurosci..

[55] Brassen S, Gamer M, Rose M, Büchel C (2010). The influence of directed covert attention on emotional face processing. Neuroimage.

[56] Güntekin B, Başar E (2014). A review of brain oscillations in perception of faces and emotional pictures. Neuropsychologia.

[57] Herrington JD, Taylor JM, Grupe DW, Curby KM, Schultz RT (2011). Bidirectional communication between amygdala and fusiform gyrus during facial recognition. Neuroimage.

[58] Jamieson AJ, Davey CG, Harrison BJ (2021). Differential modulation of effective connectivity in the brain’s extended face processing system by fearful and sad facial expressions. eNeuro.

[59] Vuilleumier P, Richardson MP, Armony JL, Driver J, Dolan RJ (2004). Distant influences of amygdala lesion on visual cortical activation during emotional face processing. Nat. Neurosci..

[60] Bickart KC, Dickerson BC, Feldman Barrett L (2014). The amygdala as a hub in brain networks that support social life. Neuropsychologia.

[61] Mears D, Pollard HB (2016). Network science and the human brain: Using graph theory to understand the brain and one of its hubs, the amygdala, in health and disease. J. Neurosci. Res..

[62] Barrett LF (2017). The theory of constructed emotion: An active inference account of interoception and categorization. Soc. Cognit. Affect. Neurosci..

[63] Lindquist KA, Jackson JC, Leshin J, Satpute AB, Gendron M (2022). The cultural evolution of emotion. Nat. Rev. Psychol..

[64] Goeleven, E., De Raedt, R., Leyman, L., & Verschuere, B. The Karolinska directed emotional faces: A validation study. *Cognition & Emotion* **22**(6), 1094–1118. 10.1080/02699930701626582 (2008).

[65] Blondel VD, Guillaume J-L, Lambiotte R, Lefebvre E (2008). Fast unfolding of communities in large networks. J. Stat. Mech..

[66] Newman MEJ (2006). Modularity and community structure in networks. Proc. Natl. Acad. Sci. U S A.

