# Supplementary material for: EEG alpha band functional connectivity reveals distinct cortical dynamics for overt and covert emotional face processing
Source: Sci Rep. 2023 Jun 19;13:9951. doi: 10.1038/s41598-023-36860-4 (PMC10279636; doi:10.1038/s41598-023-36860-4)
Supplement: Supplementary file 1 — Supplementary Table 1. [file 41598_2023_36860_MOESM1_ESM.docx]

| Destrieux Parcel | System | X coord (MNI) | Y coord (MNI) | Z coord (MNI) |
| --- | --- | --- | --- | --- |
| G_oc-temp_lat-fusifor L | Core System | -35.646 | -53.889 | -17.966 |
| G_oc-temp_lat-fusifor R | Core System | 34.63 | -52.534 | -17.037 |
| G_oc-temp_med-Lingual R | Core System | 12.425 | -67.656 | -4.46 |
| G_occipital_middle L | Core System | -39.239 | -82.374 | 11.232 |
| G_occipital_middle R | Core System | 40.033 | -79.453 | 12.821 |
| G_occipital_sup L | Core System | -14.847 | -86.955 | 26.707 |
| G_occipital_sup R | Core System | 18.948 | -85.935 | 31.252 |
| Pole_occipital R | Core System | 17.394 | -94.985 | -3.658 |
| S_collat_transv_ant L | Core System | -40.799 | -21.096 | -23.879 |
| S_oc-temp_lat R | Core System | 43.127 | -50.709 | -13.523 |
| S_oc-temp_med_and_Lingual L | Core System | -30.414 | -48.15 | -9.37 |
| S_temporal_sup L | Core System | -48.624 | -47.475 | 5.828 |
| S_temporal_sup R | Core System | 48.138 | -43.634 | 8.103 |
| G_Ins_lg_and_S_cent_ins L | Extended System | -36.46 | -7.484 | -0.058 |
| G_and_S_cingul-Ant L | Extended System | -10.877 | 39.073 | 8.334 |
| G_and_S_cingul-Ant R | Extended System | 11.187 | 38.601 | 8.006 |
| G_and_S_cingul-Mid-Ant L | Extended System | -10.191 | 13.856 | 35.766 |
| G_and_S_frontomargin L | Extended System | -24.026 | 53.836 | -6.229 |
| G_and_S_subcentral L | Extended System | -56.559 | -11.103 | 15.503 |
| G_cingul-Post-ventral R | Extended System | 10.218 | -49.055 | 7.38 |
| G_front_inf-Opercular L | Extended System | -50.085 | 11.87 | 7.46 |
| G_front_inf-Orbital R | Extended System | 47.925 | 32.35 | -6.988 |
| G_front_inf-Triangul R | Extended System | 51.05 | 28.051 | 6.786 |
| G_front_middle L | Extended System | -37.27 | 29.7 | 31.553 |
| G_front_middle R | Extended System | 38.204 | 29.401 | 30.849 |
| G_front_sup L | Extended System | -9.03 | 21.65 | 51.327 |
| G_front_sup R | Extended System | 9.28 | 22.781 | 52.457 |
| G_orbital L | Extended System | -27.36 | 30.677 | -17.425 |
| G_orbital R | Extended System | 26.58 | 30.04 | -17.02 |
| G_pariet_inf-Angular L | Extended System | -42.927 | -64.441 | 38.345 |
| G_pariet_inf-Supramar L | Extended System | -55.777 | -36.111 | 31.904 |
| G_parietal_sup L | Extended System | -18.662 | -57.73 | 58.569 |
| G_postcentral L | Extended System | -45.544 | -25.929 | 54.949 |
| G_precentral L | Extended System | -41.773 | -9.72 | 51.554 |
| G_precentral R | Extended System | 40.651 | -8.156 | 53.418 |
| G_rectus L | Extended System | -5.893 | 36.009 | -20.016 |
| G_rectus R | Extended System | 6.54 | 33.631 | -20.422 |
| G_subcallosal L | Extended System | -5.987 | 15.553 | -8.696 |
| G_subcallosal R | Extended System | 6.973 | 15.34 | -11.926 |
| G_temp_sup-G_T_transv L | Extended System | -47.781 | -21.011 | 5.17 |
| G_temporal_inf L | Extended System | -51.331 | -37.894 | -24.64 |
| G_temporal_inf R | Extended System | 52.585 | -34.688 | -23.578 |
| G_temporal_middle L | Extended System | -58.882 | -35.896 | -12.614 |
| Pole_temporal L | Extended System | -34.89 | 2.935 | -37.098 |
| Pole_temporal R | Extended System | 38.295 | 5.021 | -36.584 |
| S_central L | Extended System | -36.398 | -21.981 | 46.486 |
| S_circular_insula_ant L | Extended System | -27.738 | 22.278 | -9.516 |
| S_circular_insula_inf L | Extended System | -39.502 | -13.872 | -7.846 |
| S_front_inf L | Extended System | -38.112 | 22.769 | 21.882 |
| S_front_sup L | Extended System | -22.711 | 17.928 | 43.159 |
| S_intrapariet_and_P_trans L | Extended System | -28.528 | -58.348 | 38.653 |
| S_orbital-H_Shaped R | Extended System | 24.293 | 35.417 | -11.756 |
| S_pericallosal L | Extended System | -6.016 | -10.328 | 25.728 |
| S_pericallosal R | Extended System | 7.329 | -8.271 | 25.71 |
| S_postcentral L | Extended System | -36.815 | -36.102 | 41.146 |
| S_precentral-inf-part L | Extended System | -43.352 | 2.957 | 27.034 |
| S_precentral-inf-part R | Extended System | 42.614 | 4.307 | 27.246 |
| S_precentral-sup-part L | Extended System | -26.797 | -9.499 | 50.401 |
| S_precentral-sup-part R | Extended System | 26.621 | -8.68 | 51.56 |
| S_suborbital L | Extended System | -8.559 | 38.452 | -11.471 |

Legend

Node assignment to the two subsystems (*core* and *extended*) of the Face Processing Network, with their respective coordinates in the MNI space
